# Giant phyllodes tumor of the breast

**DOI:** 10.11604/pamj.2022.42.73.35409

**Published:** 2022-05-26

**Authors:** Suruchi Dhawan, Avinash Dhok

**Affiliations:** 1 Department of Radiodiagnosis and Imaging, Narendra Kumar Prasadrao (NKP) Salve Institute of Medical Sciences and Lata Mangeshkar Hospital, Nagpur, Maharashtra, India

**Keywords:** Phyllodes tumor, fibroepithelial tumor of breast, cystosarcoma

## Image in medicine

A 50-year-old female presented to the Outpatient Department (OPD) with chief complaints of a lump in the left breast for 3 years. There was a history of a rapid increase in the size of the lump for 1 year. No history of associated pain or discharge from the nipple was present. On examination, a sizeable nodular lump was palpated in the left breast with intact overlying skin and nipple-areola complex. The patient was advised sonomammography of the left breast, which revealed a large loculated well-circumscribed lesion of approximate size 9.3 x 8.6 x 6.7 cm. It had a heterogeneous echotexture, with solid and cystic components within the lesion. Multiple thick interconnecting septae were noted within the lesion. The cystic component had posterior acoustic enhancement with multiple dense moving internal echoes within. No calcification was present within the lesion. The solid component and septae showed vascularity on color Doppler. No evidence of axillary lymphadenopathy or invasion into the underlying muscles or bone was noted. The lesion was diagnosed as a case of benign phyllodes tumor on sonomammography due to its peculiar characteristics. The patient was advised of surgical resection of the tumor and the mass was sent for histopathological evaluation, which confirmed the diagnosis of benign phyllodes tumor. Phyllode's tumor is also known as cystosarcoma phyllodes and is a rare fibroepithelial tumor of the breast. These tumors are mostly benign and have a resemblance to fibroadenomas on imaging in the early stages. However, fibroadenomas are usually small and stop growing in size beyond 3-5 cm whereas, phyllodes tumors are large at presentation and can grow up to an enormous size.

**Figure 1:**
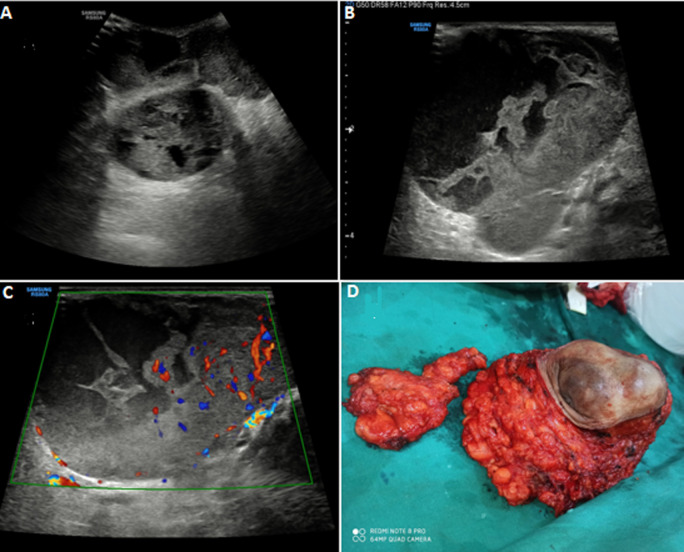
A) B-mode image using a low-frequency convex probe showing a heterogenous solid cystic lesion in left breast parenchyma; B) B-mode image using a high-frequency linear probe showing multiple echoes and septae within the cystic component; C) color Doppler image showing significant vascularity within the solid component; D) post-operative image of the resected mass

